# DNA N^6^-methyladenine modification in hypertension

**DOI:** 10.18632/aging.103023

**Published:** 2020-04-13

**Authors:** Ye Guo, Yuqing Pei, Kexin Li, Wei Cui, Donghong Zhang

**Affiliations:** 1 Department of Laboratory Medicine, Peking Union Medical College Hospital and Peking Union Medical College, Beijing 100021, PR China; 2 State Key Laboratory of Molecular Oncology, Department of Clinical Laboratory, National Clinical Research Center for Cancer/Cancer Hospital, Chinese Academy of Medical Sciences and Peking Union Medical College, Beijing 100021, PR China; 3 Center for Molecular and Translational Medicine, Georgia State University, Research Science Center, Atlanta, GA 30303, USA

**Keywords:** N6-methyladenine, hypertension, vascular smooth muscle cells, ALKBH1, HIF1α

## Abstract

DNA methylation has a role in the pathogenesis of essential hypertension. DNA N6-methyladenine (6mA) modification as a novel adenine methylation exists in human tissues, but whether it plays a role in hypertension development remains unclear. Here, we reported that the global 6mA DNA level in leukocytes was significantly reduced in patients with hypertension and was reversed with successful treatment. Age, systolic blood pressure, and serum total cholesterol and high-density lipoprotein levels were associated with decreased leukocyte 6mA DNA level. Elevated ALKBH1 (AlkB homolog 1), a demethylase of 6mA, level mediated this dynamic change in 6mA level in leukocytes and vascular smooth muscle cells in hypertension mouse and rat models. Knockdown of ALKBH1 suppressed angiotensin II-induced vascular smooth muscle phenotype transformation, proliferation and migration. ALKBH1-6mA directly and negatively regulated hypoxia inducible factor 1 α (HIF1α), which responded to angiotensin II-induced vascular remodeling. Collectively, our results demonstrate a potential epigenetic role for ALKBH1-6mA regulation in hypertension development, diagnosis and treatment.

## INTRODUCTION

Essential hypertension is the number 1 identifiable risk factor for death worldwide [1] and it affects both sexes, mainly older patients [2, 3]. This age-related condition affects about a quarter of the adult population, with severe complications. Epidemiological survey shown that 20% to 50% of the inter-individual variability in blood pressure is heritable. Genetic and epigenetic components have a prominent role in the development of essential hypertension [4]. However, the precise pathogenic mechanisms remain unknown, which limits opportunities for early prevention and effective treatment. As compared with genetic factors, changed epigenetics are reversible with the progression and treatment of hypertension [5, 6]. Thus, epigenetic measurement and therapy confers new ideas and methods for the diagnosis and treatment of hypertension.

Epigenetic modifications associated with hypertension mainly include DNA methylation, micro-RNA, noncoding RNA, and histone modifications [7–9]. Notably, 5-methylcytosine (5mC) DNA methylation is a stable and inheritable epigenetic modification. Aberrant 5mC DNA methylation is the most well-defined epigenetic modification that regulates gene transcription affecting the pathogenesis, duration, and severity of essential hypertension [10, 11]. For example, decreased global 5mC level in peripheral blood is correlated with increased essential hypertension severity [11, 12]. A genome-wide association study identified 12 genetic variants distributed in multiple CpG sites closely related to blood pressure [13]. Especially, hypomethylation of the renin-angiotensin-aldosterone system, including angiotensin receptor subtype 1a, α-adducin 1, 11-β hydroxysteroid dehydrogenase type II, adrenergic receptor and prolylcarboxypeptidase genes was correlated with essential hypertension risk and treatment outcome [14–16]. Our previous data revealed human telomerase reverse transcriptase hypomethylation in the clinical and rat model of hypertension, which contributed to shortened leukocyte telomere length [9]. However, knowledge of the association between DNA methylation and hypertension is still in its infancy.

Recently, with the development of deep sequencing, a novel DNA adenine methylation (N^6^-methyl-2’-deoxyadenosine [6mA]) was found in prokaryotes and eukaryotes. 6mA has been identified as an epigenetic mark that carries heritable epigenetic information in *Caenorhabditis elegans* [17]. Although evidence from these studies suggests potential epigenetic roles for 6mA, its precise biological function(s) remain elusive [18, 19]. N6-adenine-specific DNA methyltransferase 1 (N6AMT1) and demethylase AlkB homolog 1 (ALKBH1) were recently identified as responsible for most 6mA methyltransferase and demethyltransferase activity in human cells [20]. Recent studies demonstrated that 6mA is dynamically changed by dysregulation of N6AMT1 and ALKBH1 in human tumorigenesis [20]. 6mA participates in cancer survival and proliferation by corroborating with H3K9me3 [21, 22]. However, the roles of 6mA in human cardiovascular disease, including hypertension, are largely unknown.

In the current study, we explored the potential profile, function and clinical significance of 6mA DNA modification in patients with clinical hypertension, a hypertension model in mouse and rat, and in cultured cells. Global leukocyte 6mA DNA level was significantly reduced in hypertension and reversed by anti-hypertension treatment. ALKBH1 regulated the dynamic changes of 6mA. Knockdown of ALKBH1 suppressed angiotensin II (Ang II)-induced transformation, proliferation and migration of vascular smooth muscle cells (VSMCs) by regulating hypoxia inducible factor 1α (HIF1α). These results suggest a potential epigenetic role for 6mA in hypertension diagnosis and treatment.

## RESULTS

### Reduced leukocyte 6mA DNA in hypertension patients could recover to normal level with treatment

**Figure 1:**
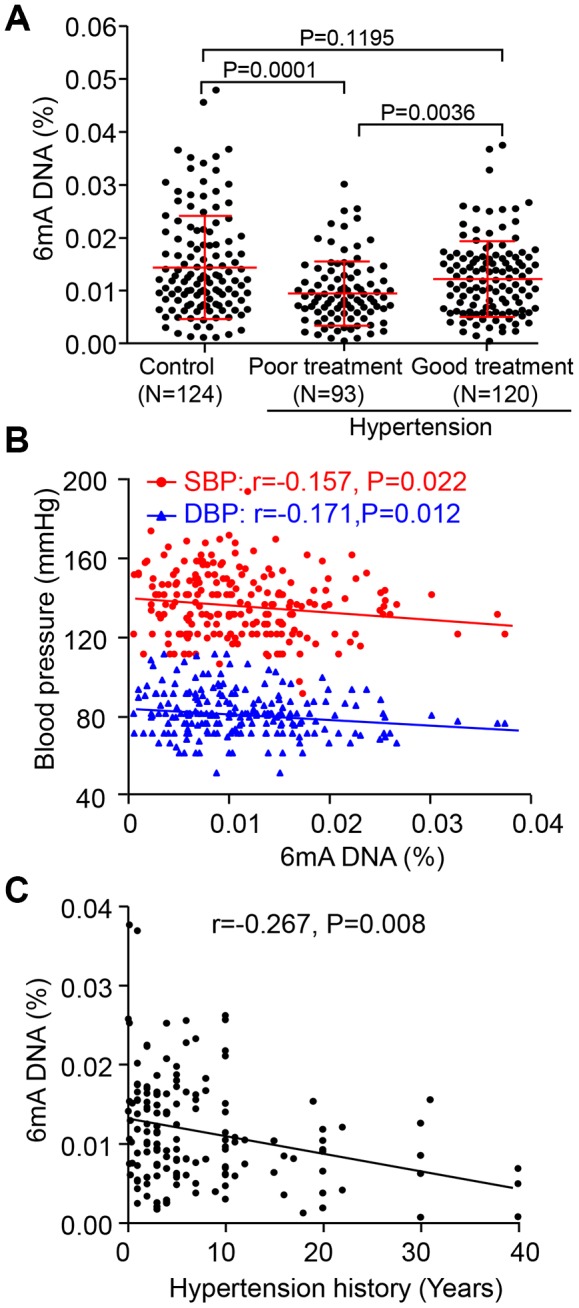
**Decreased leukocyte N6-methyladenosine (6mA) DNA level is associated with hypertension development and treatment.** (**A**) Overall leukocyte 6mA level in people with hypertension by drug treatment successful (Good) or not (Poor), as well as in the normal individuals (Control). (**B**, **C**) Spearman correlation coefficients for leukocyte 6mA level correlated with systolic blood pressure (SBP) and diastolic blood pressure (DBP), as well as hypertension history. Data are mean ± SD and were compared by unpaired *t* test for **A** and **B**.

To explore the effect of global 6mA DNA modification of leukocytes in patients with hypertension, we first found leukocyte 6mA methylation was reduced in hypertension patients with poor treatment compared with normal control subjects. Notably, 6mA has come back to the normal level by successful treatment of hypertension (Figure 1A). As well, 6mA DNA level was negatively correlated with systolic blood pressure (SBP) and/or diastolic blood pressure (DBP) in hypertension patients (Figure 1B). Patients with low 6mA DNA often have a long hypertension history (Figure 1C). However, males and females did not differ in 6mA DNA level in normal controls and hypertension patients (Supplementary Figure 1A). Individuals > 60 years old had low 6mA DNA level as compared with young men, < 55 years old, for both groups (Supplementary Figure 1B, 1C).

The relationship between 6mA DNA level and biochemical characteristics was further analyzed. Linear regression analysis showed that 6mA DNA level was inversely associated with age- and sex-adjusted SBP, DBP and levels of homocysteine, total cholesterol (TC), triglycerides (TG) and low-density lipoprotein (LDL) but positively associated with level of high-density lipoprotein (HDL) for hypertension patients (Table 1). Age, SBP and TC and HDL levels were still associated with decreased of 6mA DNA level in stepwise multivariable analysis. Thus, leukocyte 6mA DNA level could be a sensitive diagnosis and treatment biomarker for hypertension patients.

**Table 1:** Linear regression and multivariate model for the association of clinical factors and leukocyte 6mA DNA level for control participants and hypertension patients.

**Clinical factors**	**Age and sex-adjusted 6mA**		**Multivariate model**	
	**r**	**p**	**r**	**p**
Systolic blood pressure	**-0.238**	**0.005**	**-0.162**	**0.029**
Diastolic blood pressure	**-0.219**	**0.01**		
Age (years)			**-0.211**	**0.017**
Total cholesterol	**-0.291**	**0.01**	**-0.201**	**0.036**
High-density lipoprotein	**0.315**	**0.005**	**0.240**	**0.011**
Homocysteine	**-0.319**	**0.0001**		
Triglycerides	**-0.211**	**0.013**		
Low-density lipoprotein	**-0.187**	**0.028**		
Creatinine	-0.110	0.144		
Lactate dehydrogenase	-0.122	0.133		
Alanine aminotransferase	0.003	0.97		
Total bilirubin	0.115	0.181		
Direct bilirubin	0.112	0.194		
Cholinesterase	0.072	0.404		
Uric acid	-0.109	0.205		

### Elevated ALKBH1 level decreases the 6mA DNA level in leukocytes and VSMCs in the *in vivo* and *in vitro* hypertension model

**Figure 2:**
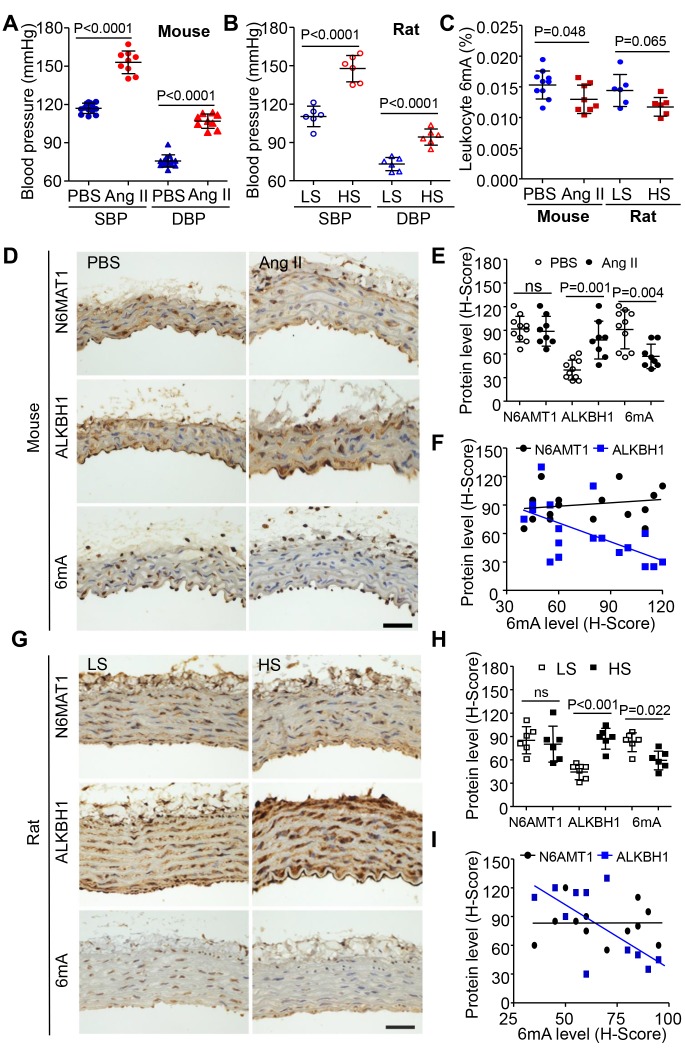
**Effect of 6mA DNA level and its modulators in leukocytes and vasculature of mice and rat hypertension models.** (**A**, **B**) Elevated SBP and diastolic blood pressure (DBP) in angiotensin II (Ang II)-infused C57BL6 wild-type mice (**A**) and Dahl salt-sensitive rats treated with high salt (HS, 8% NaCl) (**B**) compared with their controls. (**C**) Reduced 6mA DNA level in leukocytes of mice and rat hypertension models. (**D**, **E**) Representative immunohistochemistry (IHC) and quantification of ALKBH1, N6AMT1 and 6mA levels in vascular smooth muscle cells (VSMCs) of mouse thoracic aorta with sterile saline (Ctrl) or Ang II infusion. Scale bar: 50 μm. (**F**) Spearman correlation coefficient for m6A level correlated with ALKBH1 or N6AMT1 protein levels in VSMCs of mouse thoracic aorta. (**G**, **H**) Representative IHC and quantification of ALKBH1, N6AMT1 and 6mA levels in VSMCs of rat thoracic aortas with low salt (LS; 0.4% NaCl) or HS treatment. Scale bar: 50 μm. (**I**) Spearman correlation coefficient for m6A level correlated with ALKBH1 or N6AMT1 protein levels in VSMCs of rat thoracic aorta. Data are mean ± SD and were compared by unpaired *t* test in (**A**–**C**, **E** and **H**).

We next determined the regulation of 6mA in hypertension in mouse and rat models. Hypertension models were established by Ang II (1.44 mg/kg/day) infused in C57BL/6 mice and DSS (Dahl salt-sensitive) rats treated with 8% NaCl diet (high salt, HS) (Figure 2A and 2B). Consistent with the clinical investigation, leukocyte of 6mA DNA level was also reduced in both mouse and rat hypertension models (Figure 2C). Immunohistochemistry (IHC) staining revealed reduced 6mA DNA level in VSMCs not endothelial cells (ECs) of rats and mice with hypertension as compared with controls. Similarly, ALKBH1, the demethyltransferase of 6mA, was upregulated and negatively associated with 6mA DNA level in VSMCs of hypertensive mice or rats; the level of aortic N6AMT1, the methyltransferase of 6mA by hypertension, showed no change (Figure 2D–2I, Supplementary Figure 2A, 2B).

**Figure 3:**
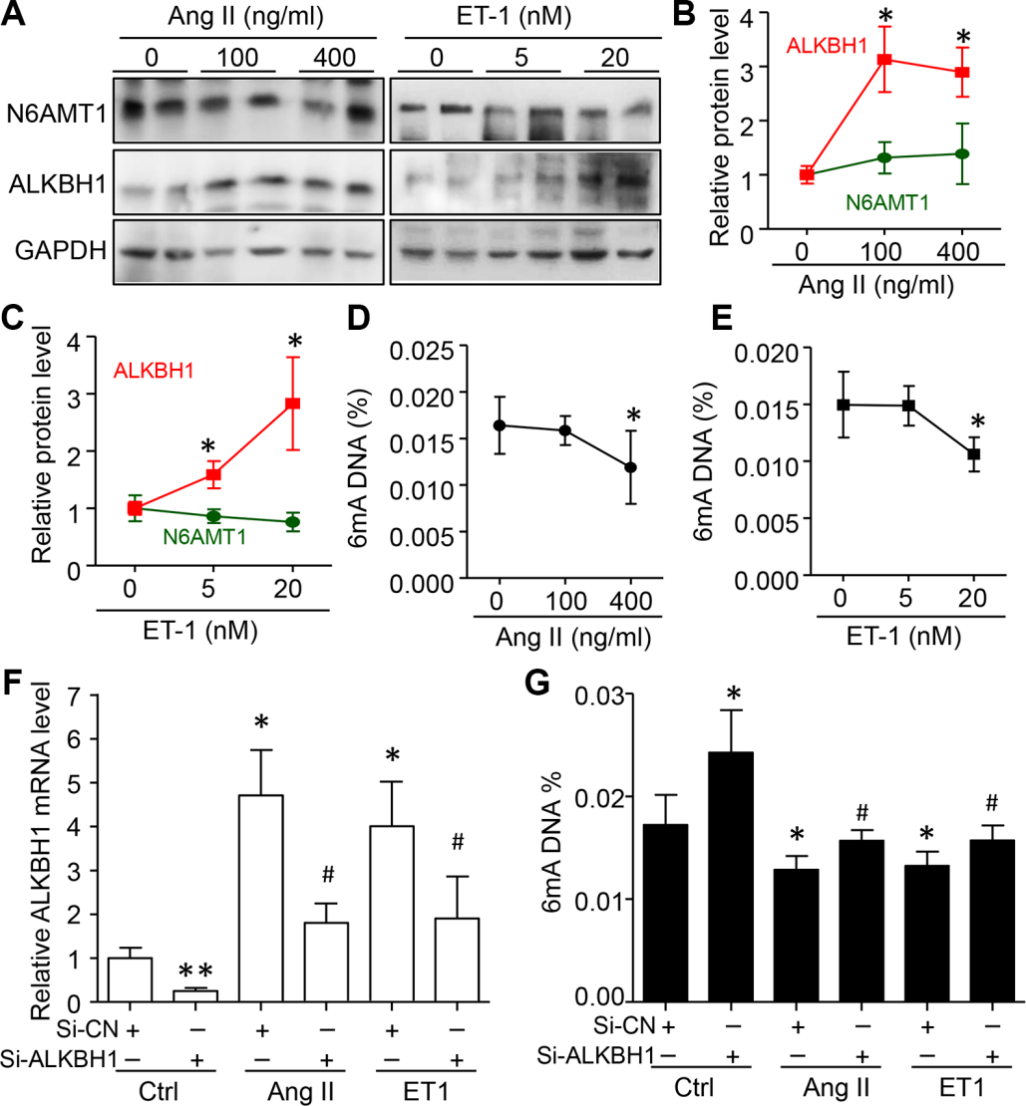
**Elevated ALKBH1 level reduced 6mA DNA level in human aortic smooth muscle cells (HASMCs).** (**A**–**C**) Representative western blot and quantification of N6AMT1 and ALKBH1 protein levels in HASMCs treated with various concentrations of angiotensin II (Ang II) or endothelin-1 (*ET1*) for 72 h. GAPDH was the internal control. (**D**, **E**) Global 6mA level measured by ELISA assay in Ang II- or *ET1*–treated HASMCs. (**F**, **G**) ALKBH1 mRNA level by RT-qPCR assay (**F**) and global m6A level by ELISA analysis (**G**) in Ang II- or *ET1*–treated HASMCs with siRNA-Control (Si-CN) or siRNA-ALKBH1 transfection. Data are mean ± SD (n= 4/group) and were analyzed by one-way ANOVA, followed by Bonferroni’s multiple comparison test. *P<0.05, **P<0.01 vs the control of Ang II or ET-1. ^#^P<0.05 vs Si-CN and Ang II or ET1 treatment for F and G.

To confirm these observations, HASMCs were treated with different concentrations of Ang II and Endothelin 1 (ET-1), two risk factors of hypertension. ALKBH1 but not N6AMT1 was dose-dependently upregulated by Ang II and ET-1 treatment (Figure 3A–3C). As predicted, with increased ALKBH1 level, global 6mA DNA level was reduced with high concentration of Ang II and ET-1 (Figure 3D, 3E). Importantly, silencing of ALKBH1 by siRNA transfection could inhibit the reduced 6mA DNA level in the basal level and in Ang II- and ET-1–treated HASMCs (Human aortic smooth muscle cells) (Figure 3F, 3G). Thus, elevated ALKBH1 in VSMCs of hypertension models was directly associated with reduced 6mA DNA level *in vivo* and *in vitro*.

### Knockdown of ALKBH1 suppresses Ang II-induced HASMCs phenotype transformation, proliferation and migration

**Figure 4:**
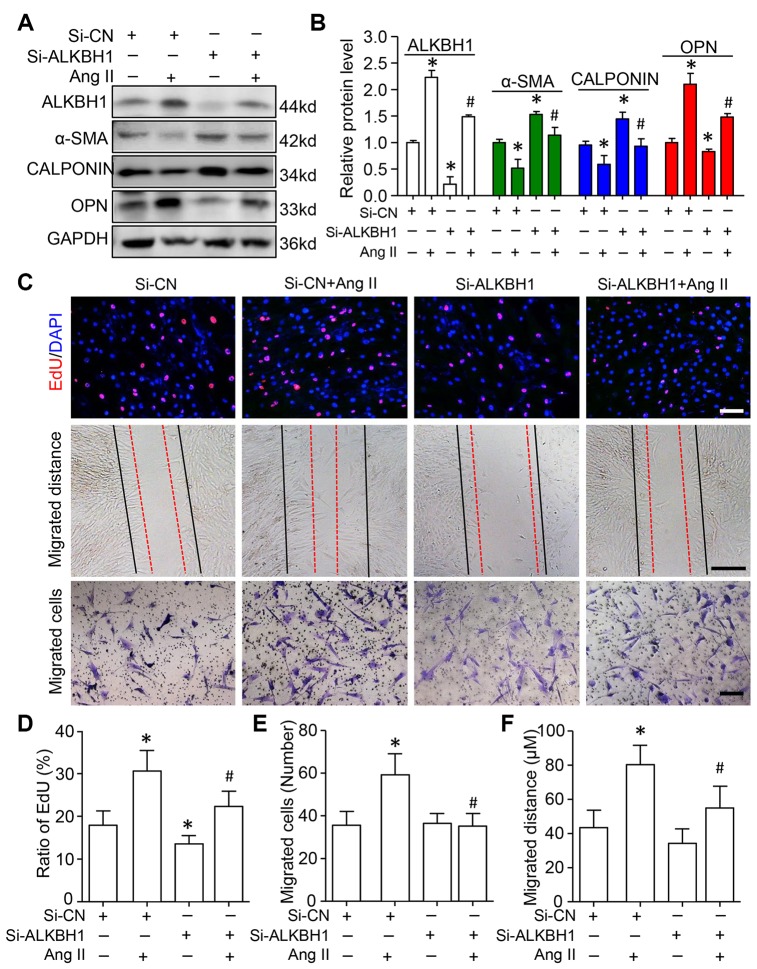
**Knockdown of ALKBH1 suppresses Ang II-induced VSMC phenotype transformation, proliferation and migration.** (**A**, **B**) Representative western blot and quantification of levels of ALKBH1, alpha-smooth muscle actin (α-SMA), CALPONIN, and osteopontin (OPN) in si-RNA-ALKBH1 (Si-ALKBH1)– or si-RNA Control (Si-CN)–transfected HASMCs with Ang II treatment or not. (**C**–**F**) Representative images of EdU staining, HASMC migration distance and number with Si-ALKBH1 and/or Ang II treatment and quantification. Scale bar: 100 μm. *P < 0.05, **P < 0.01, compared with Si-CN and no treatment of Ang II. ^#^P < 0.05 compared with Si-CN and Ang II treatment. Data are mean ± SD (n= 3/group for **A** and **B**, n= 5/group for **C**–**F**). One-way ANOVA followed by Bonferroni’s multiple comparison test was used for statistical analysis in **B** and **D**.

We assessed the function of ALKBH1 in Ang II-induced VSMC phenotype switching, cellular migration and proliferation. siRNA was transfected into HASMCs to specifically downregulate the expression of ALKBH1 before treatment with Ang II. Western blot analysis indicated increased protein expression of contractile phenotype markers (α-SMA and CALPONIN), with decreased expression of the synthetic phenotype marker (Osteopontin, OPN) on ALKBH1 silencing at the basal level. Moreover, ALKBH1 downregulation inhibited Ang II-induced VSMCs from a contractile to secretory phenotype: decreased α-SMA and CALPONIN levels and increased OPN level (Figure 4A and 4B). EdU (5-Ethynyl-2´-deoxyuridine) labeling assay revealed that cell proliferation was significantly suppressed after knocking down ALKBH1 at the basal level of VSMCs (Figure 4C and 4D). Moreover, ALKBH1 reduction could also rescue the Ang II-enhanced proliferative potential of VSMCs *in vitro*. Results from the Transwell assay and scratch test showed that downregulation of ALKBH1 could decrease the number and distance of migrated VSMCs under pathological but not physiological conditions (with or without Ang II treatment) (Figure 4C–4F). Therefore, ALKBH1 could maintain Ang II-induced vascular remodeling.

### HIF1α is a novel ALKBH1-6mA DNA modification target gene in VSMCs

**Figure 5:**
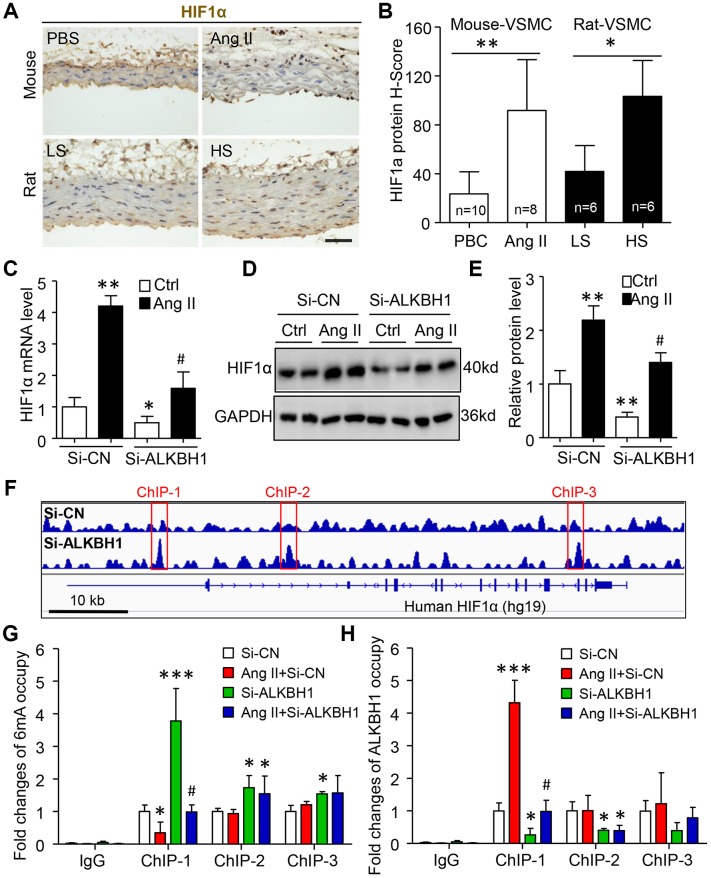
**Ang II upregulates HIF1α by ALKBH1-mediated m6A modification.** (**A**, **B**) Images and quantification of IHC staining for HIF1α in VSMCs of thoracic aortas from mice and rats with hypertension. Scale bar: 50 μm. Data as mean ± SD., *p < 0.05, **p < 0.01 by unpaired Student’s t test. (**C**–**E**) HIF1α mRNA and protein analysis by RT-qPCR and western blot assay in HASMCs with Si-ALKBH1 and/or Ang II treatment. (**F**) Integrative genomics viewer plots showing increasing m6A peaks (red-labeled ChIP-1 to -3) in human HIF1α gene (hg19) region with ALKBH1 knockdown by siRNA from previous study (GEO: GSE118093). (**G**, **H**) Chromatin immunoprecipitation (ChIP) assay with m6A (**G**) or ALKBH1 (**H**) antibody used for immunoprecipitation on HIF1α gene fragments in treated HASMCs; normal IgG was an IP control. Data are mean ± SD (n= 4/group). *P<0.05, **P<0.01, ***P<0.001 vs the Si-CN; ^#^P<0.05 vs Si-CN and Ang II treatment by one-way ANOVA followed by Bonferroni’s multiple comparison test. Ctrl, sterile saline. Ang II, angiotensin II. LS, Low salt (0.4% NaCl). HS, High salt (8% NaCl).

Given that ALKBH1 mediated 6mA DNA modification-signature hypoxia-response genes [21], we focused on HIF1α, which is required for Ang II-induced vascular remodeling [23]. On IHC, HIF1α-positive VSMCs were increased in number in two hypertension models, Ang II-infused mice and HS-diet rats, as compared with their control groups (Figure 5A, 5B). RT-qPCR and western blot assay further confirmed enhanced HIF1α mRNA and protein expression in cultured HASMCs with Ang II treatment. Notably, this elevated HIF1α level could be inhibited by silencing ALKBH1 (Figure 5C, 5D). These results suggest that Ang II-mediated HIF1α expression might be closely related to ALKBH1 in VSMCs.

Bioinformatics analysis revealed three 6mA peaks around the human HIF1α gene that were increased with ALKBH1 siRNA knockdown (Figure 5F). ChIP assay with 6mA antibody confirmed that 6mA could modify the three peaks and close response to ALKBH1 knockdown. Of note, only the occupation at the first peaks could be inhibited by Ang II stimulation and further rescued by silencing of ALKBH1 (Figure 5G). These results were negatively consistent with the ALKBH1-immunoprecipited HIF1α gene and further suggested that ALKBH1-6mA modification was involved in Ang II-induced HIF1α activation (Figure 5H).

## DISCUSSION

6mA was described as a novel epigenetics marker that discriminates a newly synthesized DNA strand from the original one in bacteria and regulates human tumorigenesis [17–21, 24]. Here, we elucidated the regulation and function of 6mA in human, mouse and rat hypertension models. Similar to 5mC DNA methylation, global 6mA leukocyte levels were significantly decreased in all hypertension models relative to normal controls. The reduced 6mA levels could be recovered to normal levels with successful hypertension treatment. Elevated ALKBH1 level was responsible for decreased 6mA DNA level in leukocytes and VSMCs of *in vivo* and *in vitro* hypertension models. Knockdown of ALKBH1 suppressed Ang II-induced VSMCs phenotype transformation, proliferation and migration. We also identified ALKBH1-mediated 6mA level directly regulating HIF1α, which is required for Ang II-induced vascular remodeling. Our study suggested that 6mA is a sensitive marker for hypertension development, diagnosis and treatment.

The abundance and distribution feature of 6mA seems to be species-specific from prokaryotes to eukaryotes and human genome [24–26]. In general, the level of 6mA is lower in humans than other eukaryotes. For example, 6mA represents 1.75% of all adenines found in *E. coli* but less than 0.02% in human tissue and cell lines [20, 21, 27], which is consistent with our current report. Importantly, we found that low level of 6mA is sensitive to hypertension development and treatment. 6mA is closely related to SBP and TC and HDL levels, risk factors of hypertension. Thus, dysregulation of lipid metabolism might contribute to changes in global 6mA DNA modification. 6mA could be involved in a network of epigenetic reprogramming, including 5mC DNA, N6-methyladenosine (m6A) RNA, and histone methylation or acetylation, as a new layer of biological regulation in cardiovascular disease, including hypertension [6, 7, 28, 29].

Consistent with findings that ALKBH1 is a demethylase for 6mA promoting stem cell differentiation and tumorigenesis [21, 27, 30], our data suggest that elevated ALKBH1 level-reduced 6mA level in leukocytes and VSMCs is involved in hypertension development in human and mouse and rat models. Biologically, knockdown of ALKBH1 suppressed Ang II-induced VSMCs phenotype transformation, proliferation and migration. Ang II is widely used to induce hypertension and vascular remodeling in animal models of hypertension [31, 32]. The current study provides evidence that the inhibitory effect of ALKBH1-mediated 6mA level has a protective role in attenuating vascular remodeling.

Elevated 6mA DNA level might function as a repressive mark in human carcinogenesis [21, 27] and murine embryonic stem cells and brain [30, 33]. Moreover, ALKBH1-regulated gene are highly associated with a hypoxia signature [21]. Of note, HIF1α, as the key transcription factor mediating cellular hypoxic responses, contributes to Ang II-induced vascular remodeling and end-organ damage in the cardiovascular system [23, 34, 35]. To elucidate the mechanisms responsible for ALKBH1-mediated 6mA level in the Ang II-related hypertension model, our bioinformatics analysis revealed that human HIF1α gene had a 6mA motif [G/C]AGG[C/T] and was regulated by ALKBH1 [20, 21]. Mechanistically, silencing of ALKBH1 released the suppressed HIF1α expression and binding by 6mA at the basal level and Ang II-treated HASMCs. Our data suggest that ALKBH1 could provide a novel therapeutic target for preventing hypertension development by epigenetic reprogramming. However, future investigations are needed to determine whether ALKBH1-specific knockin and knockout mice could directly promote and prevent vascular remodeling during hypertension development.

## CONCLUSIONS

In conclusion, leukocyte 6mA DNA level could be a sensitive diagnosis and treatment epigenetic marker in hypertension development. The dynamics of 6mA DNA level is controlled by ALKBH1. Deficiency of ALKBH1 in VSMCs inhibits Ang II-induced VSMCs phenotype transformation, proliferation and migration via a HIF1α-dependent pathway. These findings highlight ALKBH1 as a critical molecule mediating the crosstalk between 6mA level modification and HIF1 α activity during vascular remodeling, which may be a novel therapeutic target to inhibit hypertension.

## MATERIALS AND METHODS

### Patients with hypertension

This was in part a hospital-based case–control study. The clinical study protocol was approved by the Peking Union Medical College Hospital Ethics Committee and conformed to the principles in the Declaration of Helsinki. Written informed consent was obtained from all participants.

A total of 213 patients with essential hypertension was randomly recruited, with blinding, from January 2016 to January 2019 in the Department of Clinical Laboratory of Peking Union Medical College Hospital, China. Essential hypertension was diagnosed according to the 2014 American Joint National Committee Evidence-Based Guideline for the Management of High Blood Pressure in Adults. Eligible hypertension patients were diagnosed at least three times during 1 year without treatment. Then, all patients received an angiotensin receptor or calcium blocker. SBP and/or DBP were lower than normal (<140/90 mmHg) for the good control group (n=120) and consistently high for the poor control group (n=93). A total of 124 sex- and age-matched healthy controls were defined as those without a history of hypertension and with SBP < 130 mmHg and DBP < 85 mmHg.

The exclusion criteria included concomitant valvar heart disease, congenital heart disease, myocardial infarction, stroke, transient ischemic attack, any revascularization procedure, acute and chronic viral or bacterial infection, asthma, tumors, thermodynamically significant carotid or peripheral arterial disease, systemic hypertension, hypercholesterolemia, sepsis, abnormal liver and renal function or syndrome, electrolyte disturbance, chromosomal disorders and connective tissue diseases. We also excluded patients using any medications likely to affect blood pressure, including non-steroidal anti-inflammatory drugs, glucocorticoids or potassium supplements.

### Blood sampling

Peripheral blood (10 ml) was obtained from the elbow vein of all participants. The plasma was harvested from the upper layer after centrifugation and stored at -80 °C. Leukocyte cell samples were separated from EDTA-anticoagulated blood and stored at -80°C for 6mA DNA methylation analysis. All biochemical assessments were measured by routine techniques at the Clinical Laboratory Department of the Peking Union Medical College Hospital. Biochemical assessments were performed under fasting conditions in the early morning. The intra- and inter-assay coefficients of variation were <10% for all biochemical variables. The baseline demographics and detailed laboratory values are in Supplementary Table 1.

### Animal models of hypertension

Male Dahl salt-sensitive rats (DSS: SS/JrHsdMcwiCrl) and C57BL-6 wild-type mice aged 8-10 weeks were obtained from Vital River Laboratory Animal Technology (Beijing). In total, 12 rats were initially fed the AIN-76A diet with 0.4% NaCl (low salt [LS], cat. 113755; Dyets) for 2 weeks. Then, six 12-week-old rats were randomly switched to 8% NaCl diet (high salt [HS]; cat. 100078; Dyets) for 8 weeks [36]. The remaining LS rats were used as controls. C57BL6 mice aged 12 weeks were subcutaneously infused with Ang II (n=9, 1.44 mg/kg/day, Sigma) for the hypertension model or sterile saline for controls (n=10) by using osmotic minipump for 4 weeks [37]. All animals were housed in a temperature-controlled room with a 12-h light/dark cycle and free access to tap water. Overdose of pentobarbital sodium (200 mg/kg, iv) was used for euthanasia at the end of the experiment. The trial was performed in a double-blinded and randomized fashion. The experimental protocols followed the Guide for the Care and Use of Laboratory Animals of Peking Union Medical College Hospital.

### Blood pressure measurement

SBP and DBP measurements were taken by tail-cuff plethysmography (Noninvasive Blood Pressure System, CODA-HT8, CT; ALC-NIBP) according to the manufacturer’s instructions. All animals were restrained and conscious after 7-day training in a quiet room by the same person. Animals were warmed to 30°C, kept calm and allowed to rest quietly. No sedation was used.

### IHC staining

6mA level and its modulators expression in tissue was detected by IHC staining [38]. Briefly, the aortic arteries of the rats and mice were harvested and fixed with 10% formalin, dehydrated and embedded in paraffin. Then, 4-mm-thick tissue slices were cut, dried, deparaffinized and treated with a boiling citrate buffer (pH 6.0) for a total of 15 min. The slides were blocked with a peroxidase block (DAKO, Carpinteria, CA) and 2.5% horse serum for 1 h, then incubated with the primary antibodies for N6AMT1, ALKBH1, 6mA and HIF1α (dilution 1:500) at 4°C overnight, then stained with the EnVision+ Dual Link System-HRP (Dako), counterstained with hematoxylin and mounted. The histoscore (H-score) was calculated by the product of the intensity of staining (graded as: 0, no staining; 1, weak; 2, median; or 3, strong) and percentage of positive cells. The range of possible scores was from 0 to 300.

### Cell culture and si-RNA transfection

HASMCs were obtained from the American Type Culture Collection (Rockville, MD, USA). Cells were cultured in F12K Kaighn’s modification medium supplemented with 10% fetal bovine serum (FBS), 100 units/ penicillin, and 100 mg/mL streptomycin at 37°C in a humidified atmosphere containing 5% CO2. The medium was replaced with fresh medium at intervals of 3-4 days. The cells were starved for 12 h in a serum-free medium before use. Scramble siRNA (Si-CN) and siRNA-ALKBH1 (Si- ALKBH1) were synthesized by Gene Pharma (Shanghai). Plasmids at 10 nM were transfected into HASMCs by using Lipofectamine RNAiMAX Transfection Reagent (cat: 13-778-075, Invitrogen) following the manufacturer’s instructions. After 24 h, the transfected cells were treated with Ang II or endothelin-1 (ET1, cat: E7764, Sigma) or not before the next experiment.

### Western blot analysis

Treated cells were homogenized on ice-cold RIPA-Lysis and Extraction buffer (Thermo Fisher Scientific, USA) and quantified by using the BCA assay kit (Pierce, Santa Cruz, USA). Equal amounts of total protein were separated by SDS-PAGE and transferred to PVDF membranes in Trisglycine methanol buffer. They were incubated with the antibodies for N6AMT1 (ab106329), ALKBH1 (ab126596) and HIF1α (ab1) purchased from Abcam (Cambridge, MA, USA). GAPDH (14C10) antibody (Cat: 2118) was obtained from Cell Signaling Technology and used as a normalization control (Beverly, MA, USA). The bands were visualized by using the Enhanced Chemiluminescence Detection Kit (Thermo Fisher Scientific, USA).

### Real-time quantitative PCR (RT-qPCR)

Total mRNA was extracted by using Trizol reagent (Life Technologies, Gaithersburg, MD, USA). cDNA was synthesized from 1.0 μg RNA by using the First-Strand cDNA Synthesis Kit (Takara, Otsu, Shiga, Japan). PCR amplification reactions were performed in duplicate with the SYBR Green PCR Master Mix (Applied Biosystems). Quantification involved the ΔΔCT method and data were normalized to the expression of GAPDH. The sequences of primers were previously described [20].

### Cell migration assay

Cell migration was evaluated by using wound-healing and Transwell migration assays [39]. Simply, HASMCs were seeded in 6-well plates for 24 hrs and scratched by using sterile 0.2 mL pipette tips in the culture dish. Cells were washed with phosphate buffered saline and then fresh DMEM with 0.5% fetal bovine serum was added. The wound gaps were recorded by using bright-field microscopy at 0 hr and 12 hrs and measured by using Image-pro plus 6.0. The migration distance was analyzed by initial wound distance subtracting the remaining distance.

For Transwell migration assays, 1×10^5^ HASMCs transfected with si-ALKBH1 or si-CN were seeded into the upper chambers, containing a filter membrane (8-μm pore size), of 24-well Transwell plates (Corning Inc., New York, USA). The lower chambers were filled with 1% fetal bovine serum medium containing 1 μg/ml Ang II (Sigma, USA) and the plate was incubated for 12 h. Migrated cells on the bottom of the filter were fixed with 4% paraformaldehyde, then stained with a crystal violet solution (Sigma-Aldrich, USA) and imaged by bright-field microscopy.

### Cell proliferation assay

HASMCs proliferation was evaluated by EdU incorporation assay according to the manufacturer’s instructions (RiboBio, R11053.2, China). Briefly, HASMCs were treated with Ang II for 24 h and incubated with EdU for 2 h, then fixed with 4% paraformaldehyde, stained by using the EdU imaging kit, and counterstained with DAPI (Vector Laboratories). The stained sections were photographed under a Zeiss Imager M2 microscope. Positive EdU-labeled cells were quantified and normalized to the total number of DAPI-stained cells.

### Chromatin immunoprecipitation (ChIP)

ChIP was conducted as described [40, 41]. Briefly, treated cells were crosslinked with 1% formaldehyde for 10 min, which was stopped with 125 mM glycine. Then, the samples were washed, scraped and collected. Pellets were lysed with protease inhibitors. The aliquots of lysates in each chromatin solution underwent immunoprecipitation with 5 μg antibodies for 6mA (ab208577), ALKBH1 (ab126596) or IgG (ab171870, all Abcam) overnight at 4 °C. A quantitative PCR assay was used for the precipitated HIF1α genomic DNA with primers specific for 6mA and ALKBH1 binding site. The enrichment of 6mA on the HIF1α gene was normalized to total input genomic DNA. The primer sequences spanned the predicted and elevated 6mA peak by ALKBH1 knockdown within the human HIF1α genomic DNA. Primers were for ChIP-1, forward: 5’-TTGTGTCTTGATTCTTGAAAGGAAA-3’, reverse: 5’-ACGAGAACAATGGCAGCAAA-3’; ChIP-2, forward: 5’-GTTCTTTTGGCTTAGGATTGACT-3’, reverse: 5’-TGTGCTAGATAAATAAAACAACA-3’; and ChIP-3, forward: 5’-GCAGAATGCTCAGAGA AAGCG -3’, reverse: 5’-AGCTAGAAAAGCAAAACCTACTACT-3’.

### 6mA DNA measurement

6mA global DNA methylation was assessed by using the MethylFlash 6mA DNA Methylation ELISA Kit (Colorimetric) kit following the manufacturer’s instructions (Epigentek, NY). Briefly, genomic DNA was extracted from peripheral blood specimens and HASMCs were treated according to the DNeasy Blood and Tissue Kit (Qiagen, CA). The integration of genomic DNA was confirmed on agarose gel and quantified by NanoDrop spectrophotometry. The methylation of 200 ng of genomic DNA was recognized by the 6mA antibody and colorimetrically quantified by an ELISA-like reaction. Relative quantification was used to calculate the proportion of 6mA (6mA%) in genomic DNA. Methylated DNA and unmethylated DNA were incubated in strip wells as positive and negative controls. Each sample was run in duplicate.

### Statistical analysis

The mean values ± standard deviation (SD) were calculated and plotted by using GraphPad Prism 7 (GraphPad Software, CA, USA). Comparisons involved two-tailed unpaired Student *t* test. Differences between multiple groups were determined by one-way AN0VA followed by Bonferroni’s post-hoc test. Spearman correlation analysis was used to investigat10e interactions among the 6mA DNA methylation and other indexes. Linear and multivariable regression analysis was used to examine the association of biological characteristics and 6mA DNA methylation. All analyses involved use of SPSS 20.0 (SPSS Inc.). Two-tailed P <0.05 was considered statistically significant.

## Supplementary Material

Supplementary Figures

Supplementary Table 1
