# Correction for: DNA N^6^-methyladenine modification in hypertension

**DOI:** 10.18632/aging.206154

**Published:** 2024-11-30

**Authors:** Ye Guo, Yuqing Pei, Kexin Li, Wei Cui, Donghong Zhang

**Affiliations:** 1 Department of Laboratory Medicine, Peking Union Medical College Hospital and Peking Union Medical College, Beijing 100021, PR China; 2 State Key Laboratory of Molecular Oncology, Department of Clinical Laboratory, National Clinical Research Center for Cancer/Cancer Hospital, Chinese Academy of Medical Sciences and Peking Union Medical College, Beijing 100021, PR China; 3 Center for Molecular and Translational Medicine, Georgia State University, Research Science Center, Atlanta, GA 30303, USA

**Keywords:** N6-methyladenine, hypertension, vascular smooth muscle cells, ALKBH1, HIF1α

**This article has been corrected:** The authors found that the images labeled “Migrated Cells” in **Figure 4C** were inadvertently used from another project (unpublished), which was similar to this published study. This misuse of images occurred during organization of the figures. The images from both projects were very similar, as they utilized the same HASMC cells, which led to this oversight during the submission process. Fortunately, the statistical analysis for “Migrated Cells” was unaffected. Only the representative images in **Figure 4C** need to be replaced. This correction does not change the conclusion or the article.

The corrected version of **Figure 4** is provided below.

**Figure 4:**
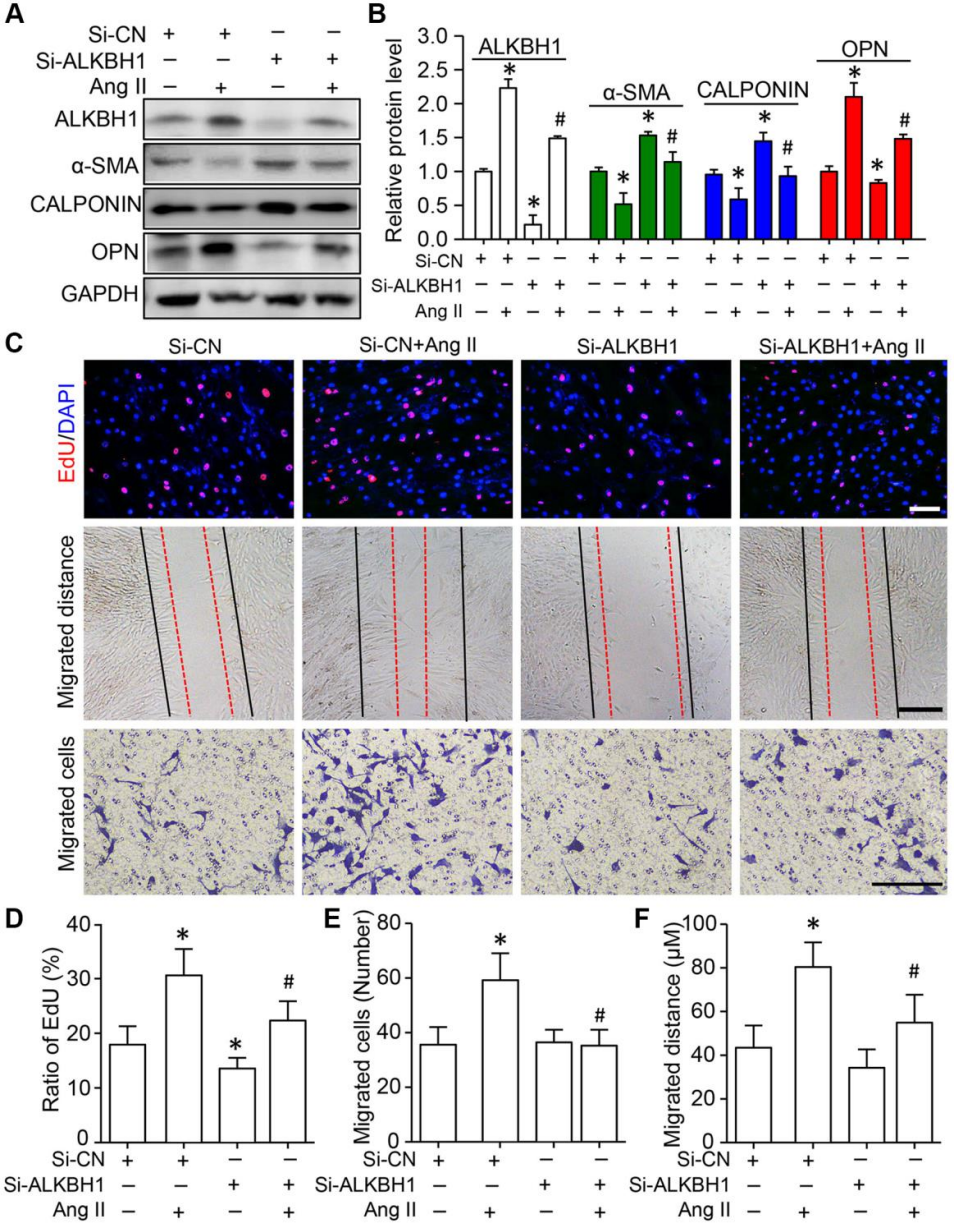
**Knockdown of ALKBH1 suppresses Ang II-induced VSMC phenotype transformation, proliferation and migration.** (**A**, **B**) Representative western blot and quantification of levels of ALKBH1, alpha-smooth muscle actin (α-SMA), CALPONIN, and osteopontin (OPN) in si-RNA-ALKBH1 (Si-ALKBH1)– or si-RNA Control (Si-CN)–transfected HASMCs with Ang II treatment or not. (**C**–**F**) Representative images of EdU staining, HASMC migration distance and number with Si-ALKBH1 and/or Ang II treatment and quantification. Scale bar: 100 µm. ^*^P < 0.05, ^**^P < 0.01, compared with Si-CN and no treatment of Ang II. ^#^*P* < 0.05 compared with Si-CN and Ang II treatment. Data are mean ± SD (*n* = 3/group for **A** and **B**, *n* = 5/group for **C**–**F**). One-way ANOVA followed by Bonferroni’s multiple comparison test was used for statistical analysis in **B** and **D**.

