# Supplementary material for: DNA N6-methyladenine modification in hypertension
Source: Aging (Albany NY). 2020 Apr 13;12(7):6276–91. doi: 10.18632/aging.103023 (PMC7185115; doi:10.18632/aging.103023)
Supplement: Supplementary Figures [file aging-12-103023-s002..pdf]

SUPPLEMENTARY FIGURES

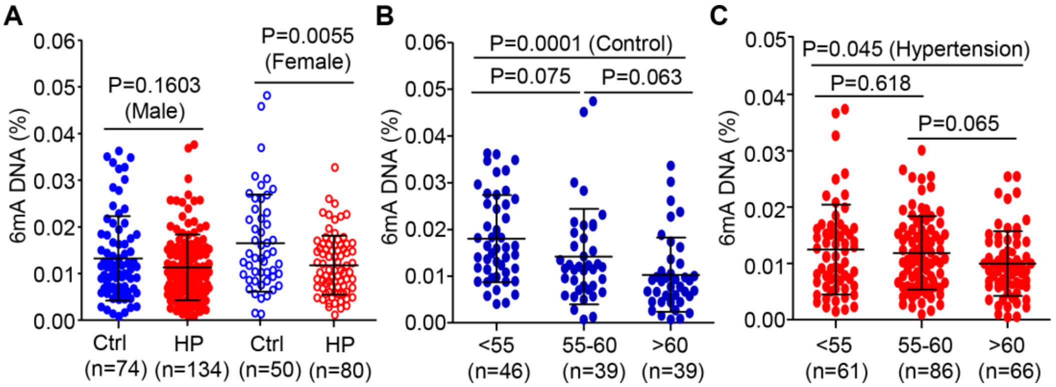

Supplementary Figure 1. Changes of leukocyte 6mA DNA by sex and age with human hypertension. (**A**–**C**) Overall leukocyte 6mA level in difference of gender and age of normal control subjects (Ctrl) and hypertension patients (HP). Data are mean ±SD and were compared by unpaired t-test.

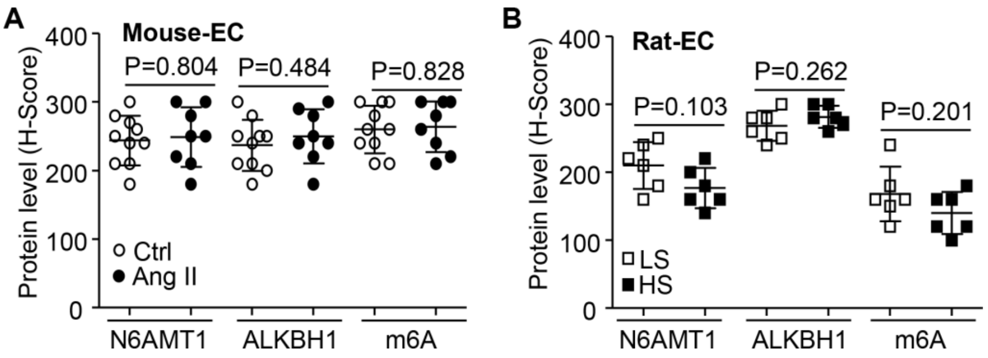

Supplementary Figure 2. Changes of m6A level and its modulators' expression in the endothelial cells (EC) of Ang II (angiotensin II) infused mouse (**A**) and HS (High salt, 8%NaCl) treated rat (**B**) hypertension model. Quantification from immunohistochemistry (IHC) staining in EC of mouse or rat thoracic aorta. Data are mean ± SD and were compared by unpaired t-test. Ctrl, sterile saline. LS, Low salt (0.4%NaCl).
