# Supplementary material for: DNA N6-methyladenine modification in hypertension
Source: Aging (Albany NY). 2020 Apr 13;12(7):6276–91. doi: 10.18632/aging.103023 (PMC7185115; doi:10.18632/aging.103023)
Supplement: Supplementary Table 1 [file aging-12-103023-s001..pdf]

SUPPLEMENTARY TABLE

Supplementary Table 1. Baseline demographics and laboratory values for normal participants and patients with poor and good hypertension control.

Clinical factors	Normal participants	Hypertensive patients (poor control)	Hypertensive patients (good control)
Systolic blood pressure (mmHg)	120.69±12.41	150.01±9.49*	123.58±9.22#
Diastolic blood pressure (mmHg)	72.25±10.40	86.94±11.60*	74.71±7.73#
Homocysteine (µM)	12.71±2.55	19.24±12.72*	16.95±13.91
Sex (Male %)	59.69	60.54	56.75
Age (Year)	61.05±7.64	62.90±14.84	62.36±13.75
Alanine aminotransferase (u/L)	24.35±15.90	26.33±21.94	25.10±13.72
Total bilirubin (µM)	11.33±4.70	12.24±4.61	13.30±544
Direct bilirubin (µM)	4.44±2.06	4.22±2.32	4.49±2.26
Lactate dehydrogenase (u/L)	175±8.72	191.72±72.37	185.70±73.64
Cholinesterase (kU/L)	8.55±3.19	15.25±3.94*	12.78±4.90
Uric acid (µM)	372±76.55	457.49±64.91*	355.84±93.24#
Total cholesterol (mM)	4.30±0.97	5.35±1.12*	4.22±1.75
Triglycerides (mM)	1.27±1.18	1.98±1.22*	1.39±1.06#
High-density lipoprotein (mM)	1.34±0.34	1.09±0.35*	1.16±0.26#
Low-density lipoprotein (mM)	2.37±0.38	2.93±0.40*	2.38±0.32#
Creatinine (µM)	73.37±17.49	141.28±70.53*	90.75±59.65#
